# Unilateral intestinal lung disease misdiagnosed as rheumatoid lung disease. A case report of absent pulmonary artery

**DOI:** 10.1016/j.rmcr.2024.101994

**Published:** 2024-02-13

**Authors:** Bimaje Akpa, Hem Desai

**Affiliations:** Department of Pulmonary, Allergy, Critical Care and Sleep Medicine, University of Minnesota Medical School, USA

**Keywords:** Unilateral ILD, Congenital absence of pulmonary artery, Rheumatoid arthritis

## Abstract

Unilateral absence of pulmonary artery (UAPA) is a congenital clinical abnormality that is rarely diagnosed in adulthood. Due to its rarity and heterogeneity as it pertains to its clinical presentation, it may be difficult to diagnose, often leading to misdiagnosis. We present a case of UAPA with unilateral pulmonary fibrosis which was misdiagnosed as rheumatoid arthritis-associated interstitial lung disease (RA-ILD). We describe the symptomology, physical examination findings, laboratory values and radiologic findings. We also describe the diagnostic challenges and approach to a patient presenting with unilateral interstitial lung disease (ILD) and highlight the importance of a comprehensive evaluation.

## 1 Introduction

Unilateral absence of pulmonary artery (UAPA) is a rare congenital abnormally caused by the failure of the sixth aortic arch to connect with the pulmonary trunk during the embryologic development [1]. It has an estimated prevalence of 1 in 200,000 [2]. It can be right or left sided. This happens when there is faulty development of sixth aortic arch in utero. System collateral branches from the aortopulmonary arteries, internal mammary artery, subclavian and intercostal arteries supply the ipsilateral peripheral pulmonary arteries [2]. Isolated UAPA involves the right lung in about two thirds of cases [3]. Given the rarity and heterogeneity as it pertains to its clinical presentation, there may be a diagnostic challenge especially given that there is no current expert consensus regarding its symptomology, diagnostic and management approach. UAPA may present as a cause of unilateral ILD usually ipsilateral. We highlight the clinical associations, review diagnostic and management principles for UAPA in adulthood. We also demonstrated the need for a high index of suspicion and comprehensive evaluation of rare causes of unilateral ILD including UAPA.

## 2 Case presentation

A 39-year-old, nonsmoker female patient with a history of hiatal hernia presented with complaints of dyspnea on exertion and joint pains in her bilateral hands, wrists, elbows for 15 years. Her joint pains appeared to be progressive and was her most bothersome symptom. Dyspnea on exertion was mild with very minimal exercise limitation. On examination, her blood pressure was 119/72 mm Hg with a pulse rate of 86 beats per minute and regular. She didn't appear to be in asny respiratory distress on general examination. She has mild tenderness over the metacarpophalangeal joints (MCP) and proximal interphalangeal joints (PIP). Breath sounds on pulmonary examination were markedly diminished on the right side with fine crackles on inspiration at the lung bases. Heart sounds were normal with no murmurs. Autoimmune work up revealed a negative positive anti-SSA antibody, positive anti-cyclic citrullinated peptide antibody (CCP) and negative rheumatoid factor. The remainder of the autoimmune profile was negative (Table 1). Thus, she was diagnosed as a case of rheumatoid arthritis (RA) as per the American College of Rheumatology Classification 2010 criteria. Due to her symptoms of shortness of breath, she underwent basic pulmonary work up.

**Table 1:** Autoimmune laboratory investigations and their results.

Laboratory Studies	Results	Ref Range/Units
CK Total	224	30–225 U/L
ESR	2	0–20 mm/hr
CRP	<2.9	0–8 mg/L
Aldolase	4.4	1.2–7.6 U/L
Scleroderma antibodies	Negative	
JO 1 Antibody IgG	Negative	
C-ANCA	<1:10	<1:10
P-ANCA	<1:10	<1:10
ANA titer	Negative	
RA Factor	8	<12 IU/mL
CCP antibody IgG	23.0	<7.0 u/ml
Anti-SS-A antibody	<0.6	<0.7 u/mL
Anti-SS-B antibody	<0.6	<0.7 u/mL
HP panel	Negative	

ANCA: Antineutrophil cytoplasmic antibodies; CCP: Cyclic citrullinated peptide; ANA: Antinuclear antibody; HP: Hypersensitivity panel; CK: Creatine kinase.

**Fig. 1:**
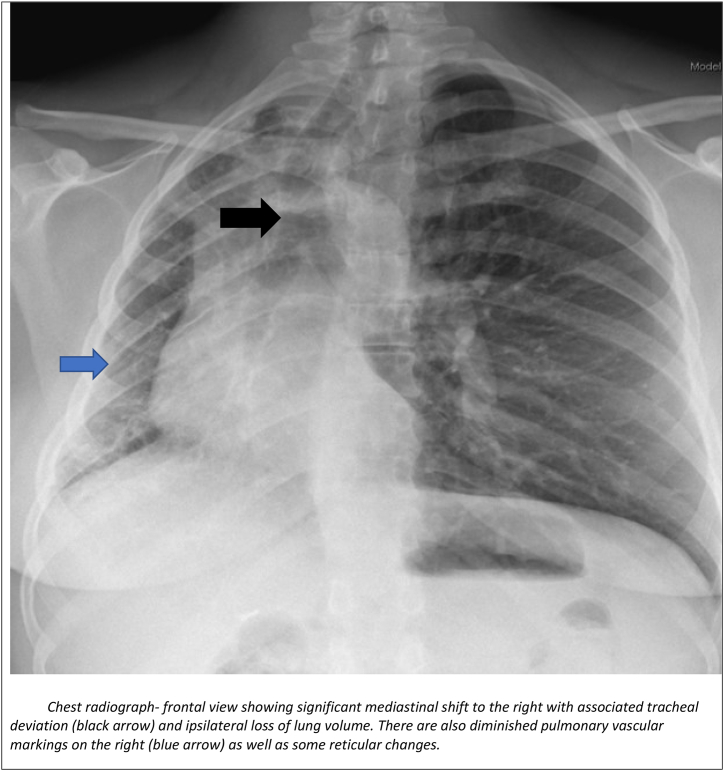
Chest radiograph-frontal view showing significant mediastinal shift to the right with associated tracheal deviation (black arrow) and ipsilateral loss of lung volume. There are also diminished pulmonary vascular markings on the right (blue arrow) as well as some reticular changes.

Chest radiography-frontal view (Fig. 1) was performed which revealed mediastinal shift to right side with volume loss on ipsilateral side and diminished pulmonary vascular markings.

**Fig. 2:**
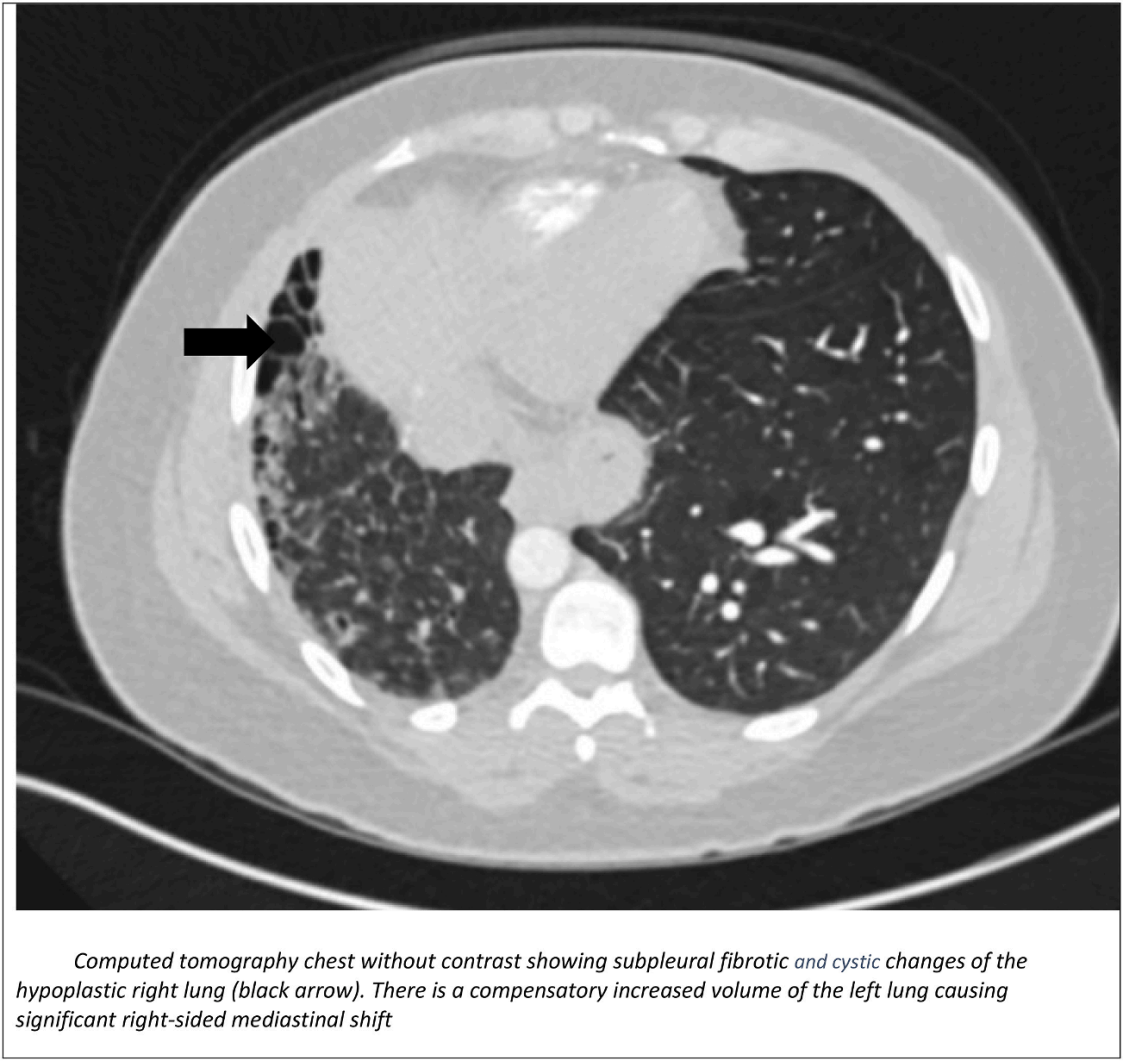
Computed tomography chest without contrast showing subpleural fibrotic and cystic changes of the hypoplastic right lung (black arrow). There is a compensatory increased volume of the left lung causing significant right-sided mediastinal shift.

Computed tomography (CT) chest without contrast (Fig. 2) did show subpleural fibrotic changes of the hypoplastic right lung with cystic changes and compensatory increased volume of the left lung causing right-sided mediastinal shift. Due to symptoms of inflammatory arthritis, autoimmune work up and findings of unilateral ILD on imaging, she was thought to have RA-ILD and was referred to rheumatology and the pulmonary clinic for consideration of immunosuppressants.

**Fig. 3:**
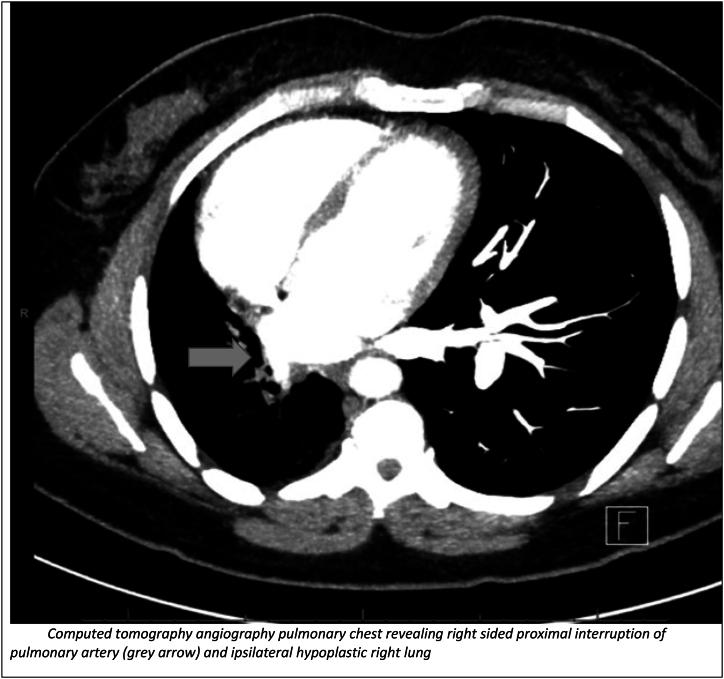
Computed tomography angiography pulmonary chest revealing right sided proximal interruption of pulmonary artery (grey arrow) and ipsilateral hypoplastic right lung.

At the pulmonary clinic, pulmonary function testing revealed normal lung volumes as well as a normal diffusion capacity. The finding of unilateral ILD was thought to be atypical for RA-ILD and prompted additional respiratory work up. Possible differentials for unilateral ILD included aspiration (since she was high risk due to her history of hiatal hernia), UAPA and radiation pneumonitis. She had never undergone radiation therapy. A swallow study with video fluoroscopy was performed which showed no evidence of aspiration and revealed normal swallow function. However, a CT pulmonary angiography (Fig. 3) revealed right sided proximal interruption of pulmonary artery and ipsilateral hypoplastic right lung and a diagnosis of UAPA was made. Further considerations were made for additional evaluations such as a 2D echocardiogram to evaluate for pulmonary hypertension (a potential complication) to help figure out if treatment for UAPA was warranted. Due to her very mild pulmonary symptoms, normal pulmonary function and discussions with patient, further investigations were not pursued. Potential complications such as recurrent chest infections, hemoptysis and pulmonary hypertension were discussed with the patient and possible need for further work up and treatments if these occurred in the future. She was advised to follow up with rheumatology for treatment of RA.

## 3 Discussion

The absence of pulmonary artery at its origin from the main pulmonary artery is known as proximal interruption of pulmonary artery (UAPA) [1]. It has an estimated prevalence of 1 in 200,000 young adults [2]. It usually occurs in conjunction with cardiovascular abnormalities such as tetralogy of Fallot or cardiac septal defects, but it can also occur in an isolated manner. Isolated UAPA involves the right lung in about two thirds of cases [3]. This happens when there is faulty development of sixth aortic arch in utero. System collateral branches from the aortopulmonary arteries, internal mammary artery, subclavian and intercostal arteries supply the ipsilateral peripheral pulmonary arteries.

Unilateral interstitial lung disease is a rare pulmonary condition associated with proximal interruption of the pulmonary artery, chronic aspiration, ipsilateral single-lung ventilation, or radiation pneumonitis [[4], [5], [6]]. A high degree of suspicion is usually needed for diagnosis of UAPA as there is some heterogeneity as it pertains to its clinical presentation. The presentation varies from dyspnea, exercise, recurrent chest infections, pulmonary hypertension, hemoptysis, and respiratory failure. These cases may be misdiagnosed as related to systemic causes such as inflammatory arthritis or other possible differentials for unilateral ILD. In our patient, she did have concomitant polyarthralgia and was thought to have RA-ILD. However, unilateral ILD is an atypical finding in RA-ILD which prompted further clinical and radiological evaluations leading to the revision of her diagnosis to UAPA. Although she was high risk for aspiration due to history of hiatal hernia, her swallow study was normal. In UAPA, chest radiography shows ipsilateral volume loss with diaphragmatic elevation, shift of heart and mediastinum to the affected side. The contralateral lung is usually shifted to the affected side. The findings of fine non-branching linear opacities at the lung periphery on chest imaging should prompt the clinician about UAPA as these are usually suggestive of vascular changes representing enlarged intercostal and *trans*-pleural pulmonary arteries. A CT pulmonary angiography is usually confirmatory and usually reveals absence of affected pulmonary artery at its origin [3]. Main differential to this radiologic finding is Swyer- James syndrome which shows air trapping on expiration not usually seen in UAPA. Treatment of UAPA in adults is based on severity of pulmonary symptoms and presence of complications. Complications include massive hemoptysis due to the rupture of collateral vessels and may require embolization otherwise the management is usually conservative [2,[7], [8], [9]]. Pneumonectomy may be needed to manage recurrent or massive hemoptysis and persistent respiratory infection [2,10].Early revascularization may allow the affected lung to develop more normally if diagnosed in early stages [[3], [8]]. In older patients, revascularization is more difficult due to significant fibrosis and narrowing of the distal pulmonary arteries. Pulmonary hypertension is another complication of UAPA and may require standard anti-pulmonary hypertension treatment such as oral phosphodiesterase inhibitors or endothelin receptor antagonist in patients with severe disease [3,8].

In our patient, further work up nor specific treatments for UAPA were not indicated since her pulmonary symptoms were mild, pulmonary function was normal and there were no clinical findings of potential complications. This was an unusual presentation as she appeared to have RA and could have been easily misdiagnosed as RA-ILD. Most previously published case reports appear to have pulmonary symptoms as their chief complaints prompting additional work up. However, in our patient, polyarthralgia related to RA was the chief complaint and the atypical finding of unilateral ILD prompted additional radiologic investigations for UAPA.

## 4 Conclusion

This case highlights the diagnostic challenges that may be faced in patients presenting with atypical presentations of pulmonary fibrosis and the high index of suspicion and investigations that are necessary when considering the possible etiologies. In adults, UAPA remains a rare entity and may be difficult to diagnose, moreover, it may present in several ways and therapy should be tailored to the patient's clinical presentation.

## CRediT authorship contribution statement

**Bimaje Akpa:** Conceptualization, Validation, Writing – original draft, Writing – review & editing. **Hem Desai:** Writing – original draft, Writing – review & editing.

## Declaration of competing interest

No conflict.
